# Uncomplicated Monochorionic Diamniotic Twins and the Timing of Delivery

**DOI:** 10.1371/journal.pmed.0020180

**Published:** 2005-06-28

**Authors:** Jane Cleary-Goldman, Mary E D'Alton

## Abstract

Cleary-Goldman and d'Alton discuss the implications of a new study in PLoS Medicine examining the risk of fetal death in uncomplicated monochorionic diamnotic twin pregnancies.

In the past 20 years, advancing maternal age and greater reliance on assisted-conception techniques have dramatically increased the incidence of multiple births, including monochorionic twins (see Glossary) [1,2]. Concomitantly, there have been advances in our understanding of these high-risk pregnancies.

**Glossary**

**Diamniotic:** Twins with two amniotic sacs.

**Monochorionic:** Twins that share one placenta.

**Monochorionic diamniotic:** Twins that share one placenta with two amniotic sacs.

**Monoamnionicity:** Twins that share one placenta and one amniotic sac.

**Twin-to-twin transfusion syndrome (TTTS):** This occurs in monochorionic twins. Twins with TTTS have vascular connections that result in unequal sharing of blood. The smaller twin (donor) does not get enough blood, while the larger twin (recipient) becomes volume overloaded.

**Intrauterine fetal demise:** This occurs when a fetus dies in utero (before birth). This is also known as stillbirth.

**Multicystic encephalomalacia:** This refers to the formation of multiple cystic lesions in the cerebral cortex of fetuses, neonates, and infants following injury usually secondary to a hypoxic event.

**Twin reversed arterial perfusion sequence:** This occurs in monochorionic twins. In this disease, there is a normally developing twin (pump twin) and a twin that does not develop a normal heart (acardiac twin). The pump twin pumps blood for the acardiac twin and for itself by reversing the blood through the umbilical artery. The pump twin is at risk for heart failure due to the increased demands on its heart.

**Nuchal translucency:** This describes the area in the back of the fetal neck during ultrasound between ten and 14 weeks' gestation. Nuchal translucency screening can be used as a method of first-trimester screening for fetal aneuploidy.

**Nonstress test:** A test assessing fetal well-being. It involves attaching fetal heart rate and uterine contraction monitors around the patient's abdomen for approximately 20 to 30 minutes and recording the fetal heart-rate patterns. A reactive nonstress test suggests fetal well-being.

## Risks of Multiple Pregnancy

The offspring of multiple pregnancies are at greater risk for adverse perinatal outcomes compared to their singleton counterparts, predominantly due to increased risks for preterm delivery and due to monochorionicity [3]. The link between monochorionicity and adverse perinatal outcomes has become increasingly strong [4]. Monochorionic pregnancies have an approximately 15% risk of developing the twin-to-twin transfusion syndrome (TTTS), which can be associated with perinatal mortality and morbidity despite treatment [5]. In addition, single intrauterine fetal demise (IUFD) in a monochorionic pregnancy may be associated with a more-than 20% risk for multicystic encephalomalacia in the surviving co-twin [6].

## Managing High-Risk Pregnancies

Despite these advances in knowledge about risks, there is very little consensus about the management of these high-risk pregnancies. In addition, there is the temptation to be reassured by increasing gestational age as the potential complications of prematurity recede. Recent studies suggest, however, that the offspring of a multiple gestation may benefit from delivering prior to their expected date of delivery [7,8].

Several studies have focused on the “prospective risk of fetal death” to help determine by which gestational age a multiple pregnancy should be delivered [7,8]. For twins, the prospective risk of fetal death appears to be equivalent to that of post-term singletons at approximately 37 to 38 weeks' gestation [7,8]. The prospective risk of fetal death for twins intersects with neonatal death at approximately 39 weeks' gestation, indicating that it may be reasonable to consider delivery of uncomplicated twins prior to 40 weeks' gestation [8]. These studies, however, did not address the impact of chorionicity on the decision to deliver a multiple pregnancy.

## A New Study of Monochorionic Twins

In this issue of *PLoS Medicine*, Barigye et al. report the prospective risk of fetal death in uncomplicated monochorionic diamniotic twin pregnancies derived from a cohort of pregnancies that were managed at a single tertiary care referral center and that delivered after 24 weeks' gestation [9]. Patients were excluded from the study if the pregnancy was complicated by TTTS, monoamnionicity, intrauterine growth restriction, growth discordance, structural anomalies, or twin reversed arterial perfusion sequence. Conjoined twins and high-order multiples were also excluded. All patients with uncomplicated monochorionic twins were managed according to a standard protocol. They received first-trimester nuchal translucency assessment and chorionicity determination, a midtrimester anatomical survey, and fetal echo followed by growth scans, amniotic fluid assessment, and Doppler studies every two weeks. The rate of fetal death was derived for two-week periods starting at 24 weeks' gestation. The prospective risk of fetal death was calculated by determining the number of IUFDs that occurred within the two-week block divided by the number of continuing uncomplicated monochorionic twin pregnancies during that same time period.

There were ten unexpected deaths (three double IUFDs and four single IUFDs) in a total of seven (4.6%) of the 151 seemingly uncomplicated monochorionic diamniotic pregnancies. These IUFDs occurred at a median gestational age of 34 weeks 1 day (range 28 weeks 0 days to 36 weeks 3 days). Between 24 and 34 weeks' gestation, the prospective risk of fetal death varied between 1/22 and 1/30 pregnancies. After 32 weeks' gestation, the prospective risk of unexpected antepartum stillbirth was 1/23. In six out of the seven pregnancies, the fetal death was incidental and found on routine ultrasound. (One case presented with decreased fetal movement.) There were no significant differences between the IUFD-affected pregnancies and the unaffected pregnancies with regards to antenatal indicators of intrauterine growth restriction and TTTS. In three of the five pregnancies (autopsy was declined in two cases), deaths remained unexplained after autopsy. Two cases, both double IUFDs, exhibited signs of TTTS. The authors concluded that despite intensive fetal surveillance, uncomplicated monochorionic diamniotic twin pregnancies are at risk for unexpected intrauterine death. The deaths occurred in the third trimester and predominantly after 32 weeks' gestation. As a result, the authors suggested that after 32 weeks' gestation, the prospective risk for fetal death in these pregnancies might be eliminated by elective preterm delivery.

## Implications of the Study

Despite the limitations of the study, which Barigye et al. elucidate well (small numbers, retrospective nature, lack of non-monochorionic twin comparative data), this study highlights an important question that many practicing obstetricians are confronting increasingly often: *when is the ideal gestational age to deliver apparently uncomplicated monochorionic twins?* The authors suggest that 32 weeks' gestation may be reasonable. At that gestational age, many of the risks associated with prematurity, such as intraventricular hemorrhage, necrotizing enterocolitis, and respiratory distress syndrome, have abated.

Nonetheless, the risks of prematurity are not negligible at 32 weeks' gestation. Balancing the risk of iatrogenic preterm birth in an *apparently* uncomplicated monochorionic twin pregnancy with the risk of double IUFD or single IUFD with the concomitant risk of multicystic encephalomalacia for the surviving co-twin is challenging. In our practice, we have been conducting antenatal surveillance more frequently than once every two weeks, and we have been using the nonstress test in addition to ultrasound and Doppler studies. Although in our preliminary experience we have not had any unexplained IUFDs, we are uncertain if the frequency of testing could account for the prospective risk of fetal death found in the study by Barigye et al. In addition, we have been offering delivery of these *apparently* uncomplicated monochorionic twins at approximately 34 to 35 weeks' gestation following antenatal corticosteroid administration and thorough counseling regarding the risks of expectant management versus elective preterm delivery. While we acknowledge that our practice pattern is by no means a standard-of-care requirement, we feel it is a reasonable approach to this dilemma until larger, prospective observational studies have been conducted to better elucidate the natural history of these high-risk pregnancies and to better answer the question of when the ideal gestational age is to deliver apparently uncomplicated monochorionic twins.

## Abbreviations

IUFD: intrauterine fetal demise
TTTS: twin-to-twin transfusion syndrome.

## References

[pmed-0020180-b1] Martin JA, Hamilton BE, Sutton PD, Ventura SJ, Menacher F (2003). Births: Final data for 2002. Natl Vital Stat Rep.

[pmed-0020180-b2] Blickstein I (2005). Estimation of iatrogenic monozygotic twinning rate following assisted reproduction: Pitfalls and caveats. Am J Obstet Gynecol.

[pmed-0020180-b3] Bajoria R, Kingdom J (1997). The case for routine determination of chorionicity and zygosity in multiple pregnancy. Prenat Diagn.

[pmed-0020180-b4] Dube J, Dodds L, Armson BA (2002). Does chorionicity or zygosity predict adverse perinatal outcomes in twins?. Am J Obstet Gynecol.

[pmed-0020180-b5] Fisk NM, Galea P (2004). Twin-twin transfusion—As good as it gets?. N Engl J Med.

[pmed-0020180-b6] Weiss JJ, Cleary-Goldman J, Budorick N, Tanji K, D Alton ME (2004). Multicystic encephalomalacia after first trimester intrauterine fetal demise in monochorionic twins. Am J Obstet Gynecol.

[pmed-0020180-b7] Sairam S, Costeloe K, Thilaganathan B (2002). Prospective risk of stillbirth in multiple-gestation pregnancies: A population based analysis. Obstet Gynecol.

[pmed-0020180-b8] Kahn B, Lumey LH, Zybert PA (2003). Prospective risk of fetal death in singleton, twin, and triplet gestations: Implications for practice. Obstet Gynecol.

[pmed-0020180-b9] Barigye O, Pasquini L, Galea P, Chambers H, Chappell L (2005). High risk of unexpected late fetal death in monochorionic twins despite intensive ultrasound surveillance: A cohort study. PLoS Med.

